# Disruption of circadian rhythm by alternating light‐dark cycles aggravates atherosclerosis development in APOE*3‐Leiden.CETP mice

**DOI:** 10.1111/jpi.12614

**Published:** 2019-10-10

**Authors:** Maaike Schilperoort, Rosa van den Berg, Laura A. Bosmans, Bram W. van Os, Martijn E. T. Dollé, Noortje A. M. Smits, Teun Guichelaar, Debbie van Baarle, Lotte Koemans, Jimmy F. P. Berbée, Tom Deboer, Johanna H. Meijer, Margreet R. de Vries, Dianne Vreeken, Janine M. van Gils, Ko Willems van Dijk, Linda W. M. van Kerkhof, Esther Lutgens, Nienke R. Biermasz, Patrick C. N. Rensen, Sander Kooijman

**Affiliations:** ^1^ Division of Endocrinology Department of Medicine Leiden University Medical Center Leiden The Netherlands; ^2^ Einthoven Laboratory for Experimental Vascular Medicine Leiden The Netherlands; ^3^ Department of Medical Biochemistry Amsterdam Cardiovascular Sciences Amsterdam University Medical Centre University of Amsterdam Amsterdam The Netherlands; ^4^ Centre for Health Protection National Institute for Public Health and the Environment Bilthoven The Netherlands; ^5^ Department of Molecular Medicine University of Texas Health Science Center at San Antonio San Antonio TX USA; ^6^ Center for Infectious Disease Control National Institute for Public Health and the Environment Bilthoven The Netherlands; ^7^ Department of Molecular Cell Biology Laboratory for Neurophysiology Leiden University Medical Center Leiden The Netherlands; ^8^ Department of Surgery Leiden University Medical Center Leiden The Netherlands; ^9^ Division of Nephrology Department of Medicine Leiden University Medical Center Leiden The Netherlands; ^10^ Department of Human Genetics Leiden University Medical Center Leiden The Netherlands; ^11^ Institute for Cardiovascular Prevention (IPEK) Ludwig‐Maximilians‐Universität Munich Germany

**Keywords:** atherosclerosis, chemokines, circadian rhythm, inflammation, monocytes

## Abstract

Disruption of circadian rhythm by means of shift work has been associated with cardiovascular disease in humans. However, causality and underlying mechanisms have not yet been established. In this study, we exposed hyperlipidemic APOE*3‐Leiden.CETP mice to either regular light‐dark cycles, weekly 6 hours phase advances or delays, or weekly alternating light‐dark cycles (12 hours shifts), as a well‐established model for shift work. We found that mice exposed to 15 weeks of alternating light‐dark cycles displayed a striking increase in atherosclerosis, with an approximately twofold increase in lesion size and severity, while mice exposed to phase advances and delays showed a milder circadian disruption and no significant effect on atherosclerosis development. We observed a higher lesion macrophage content in mice exposed to alternating light‐dark cycles without obvious changes in plasma lipids, suggesting involvement of the immune system. Moreover, while no changes in the number or activation status of circulating monocytes and other immune cells were observed, we identified increased markers for inflammation, oxidative stress, and chemoattraction in the vessel wall. Altogether, this is the first study to show that circadian disruption by shifting light‐dark cycles directly aggravates atherosclerosis development.

## 1 INTRODUCTION

Epidemiological studies have repeatedly shown associations between disturbance of biological clock function, responsible for generating circadian (ie, ~24‐hours) rhythms, and metabolic disorders such as obesity, type 2 diabetes, and cardiovascular disease (CVD).^1^, ^2^, ^3^ Already in 1949, a Scandinavian observational study among factory workers reported an association between shift work and cardiovascular mortality^4^ Longitudinal studies indicate that shift work is indeed a risk factor for cardiovascular events, including hard end‐points like ischemic stroke and myocardial infarction^5^, ^6^ However, the underlying mechanisms remained elusive. Meanwhile, the behavioral patterns of human activity, especially in industrialized countries, have undergone dramatic changes with respect to adherence to day‐night rhythms. The use of electrical light and the current 24‐hour economy have uncoupled the behavioral active period from the natural occurring day. Of note, in Europe approximately 20% of the working population is involved in some form of shift work,^7^ and in the United States and Asia, these percentages have increased to 30% and 40%, respectively.^8^, ^9^

Shift work contributes to adverse health outcomes via multifactorial pathways, including psychosocial factors, sleep loss, a decrease in physical activity, altered food intake quantity (ie, an increase in caloric intake) and quality (ie, changes in timing and choice of food), and mistimed light exposure. Aberrant light exposure disturbs the suprachiasmatic nucleus (SCN) of the hypothalamus,^10^, ^11^ which is the central pacemaker that synchronizes rhythm in peripheral organs. Additionally, physical activity and timing of food intake can act as important time cues to affect rhythm directly in peripheral organs.^12^ Thus, a combination of these factors could disrupt circadian rhythm in shift workers, resulting in CVD.

The main cause of CVD is atherosclerosis, to which dyslipidemia and a pro‐inflammatory state are key contributors. Plasma levels of lipids display day‐night variations independent of food intake,^13^ suggesting that the biological clock is an important regulator of lipid metabolism. This is supported by genetic models, showing that a defective core clock will lead to, among others, obesity.^14^ In addition, levels of immune cells and pro‐inflammatory cytokines show daily fluctuations,^15^ and functionality of the immune system has been linked to the biological clock.^16^, ^17^ Consequently, disturbed biological clock function may contribute to atherosclerosis risk through the development of dyslipidemia and a pro‐inflammatory state. The aim of the present study was to investigate whether circadian disruption through modeled shift work affects atherosclerosis development and elucidate underlying mechanisms.

## 2 MATERIALS AND METHODS

### 2.1 Experimental animals

Mice heterozygous for the APOE*3‐Leiden gene were crossbred with mice homozygously expressing human cholesteryl ester transfer protein (CETP) to yield heterozygous APOE*3‐Leiden.CETP transgenic mice,^18^ a mouse model with a human‐like lipoprotein metabolism.^19^ Female APOE*3‐Leiden.CETP mice of 8‐ to 12‐week old were fed ad libitum with a Western‐type diet (WTD) containing 15% fat from cocoa butter, 1% fat from corn oil (diet T, Altromin), enriched with 0.1% cholesterol. During a run‐in period of 3 weeks, mice were housed under standard 12 h:12 h light:dark (LD) conditions. Afterward, mice were divided over the experimental conditions by stratified randomization, to ensure similar fasting plasma total cholesterol, body weight, and age in all groups at baseline. Mice were subjected to either a regular light‐dark cycle (LD), a 6 hours phase advance every week (advance), a 6 hours phase delay every week (delay), or weekly alternating light‐dark cycles (12 hours shifts; LD‐DL) for the total duration of 15 weeks (n = 15/group). We subjected another batch of mice to either regular LD or LD‐DL (n = 34/group) for 10 weeks to gain more insight in immune cell populations and monocyte function, and to measure oxidative stress and inflammatory markers in the aortic root. After this period, mice were killed by CO_2_ inhalation and organs were collected for further analysis. Mice were group‐housed at 21°C in clear plastic cages (n = 3‐5/cage), placed in light‐tight cabinets fitted with diffuse white fluorescent light. The light intensity was verified in the animal gaze direction with an AvaSpec 2048‐SPU (Avantus BV) light meter. The spectral power distribution of the light source is shown in Figure S1. All mouse experiments were performed in accordance with the Institute for Laboratory Animal Research Guide for the Care and Use of Laboratory Animals after having received approval from the Central Animal Experiments Committee (“Centrale Commissie Dierproeven”).

For all methods of analysis used in these studies, an expanded Materials and Methods section is available in the Supporting Information, which includes information on plasma lipid measurements, behavioral analysis, histological and immunohistochemical analysis, flow cytometry, monocyte characterization and migration, and gene expression analysis.

### 2.2 Statistical analysis

All data are expressed as means ± SEM. Statistical analysis was performed using GraphPad Prism (version 7.02). Means were compared using two‐tailed unpaired Student's *t* test, one‐way ANOVA, or two‐way ANOVA followed by Dunnett's post hoc test where appropriate. When measurements were taken over time, comparisons were made using repeated measurement ANOVA, or mixed models ANOVA in case of missing values. Pearson correlation analysis was performed to examine potential linear relationships between variables. To determine the timing of the maximum in the oscillations of circulating leukocytes and plasma cholesterol, a fitting function (cosinor method) with a 24‐hours period was applied,^20^ and the obtained acrophase and corresponding *P*‐values are reported. Differences between groups were considered statistically significant if *P* < .05 (*), *P* < .01 (**), or *P* < .001 (***).

## 3 RESULTS

### 3.1 Weekly light shifts increase atherosclerosis development

**Figure 1:**
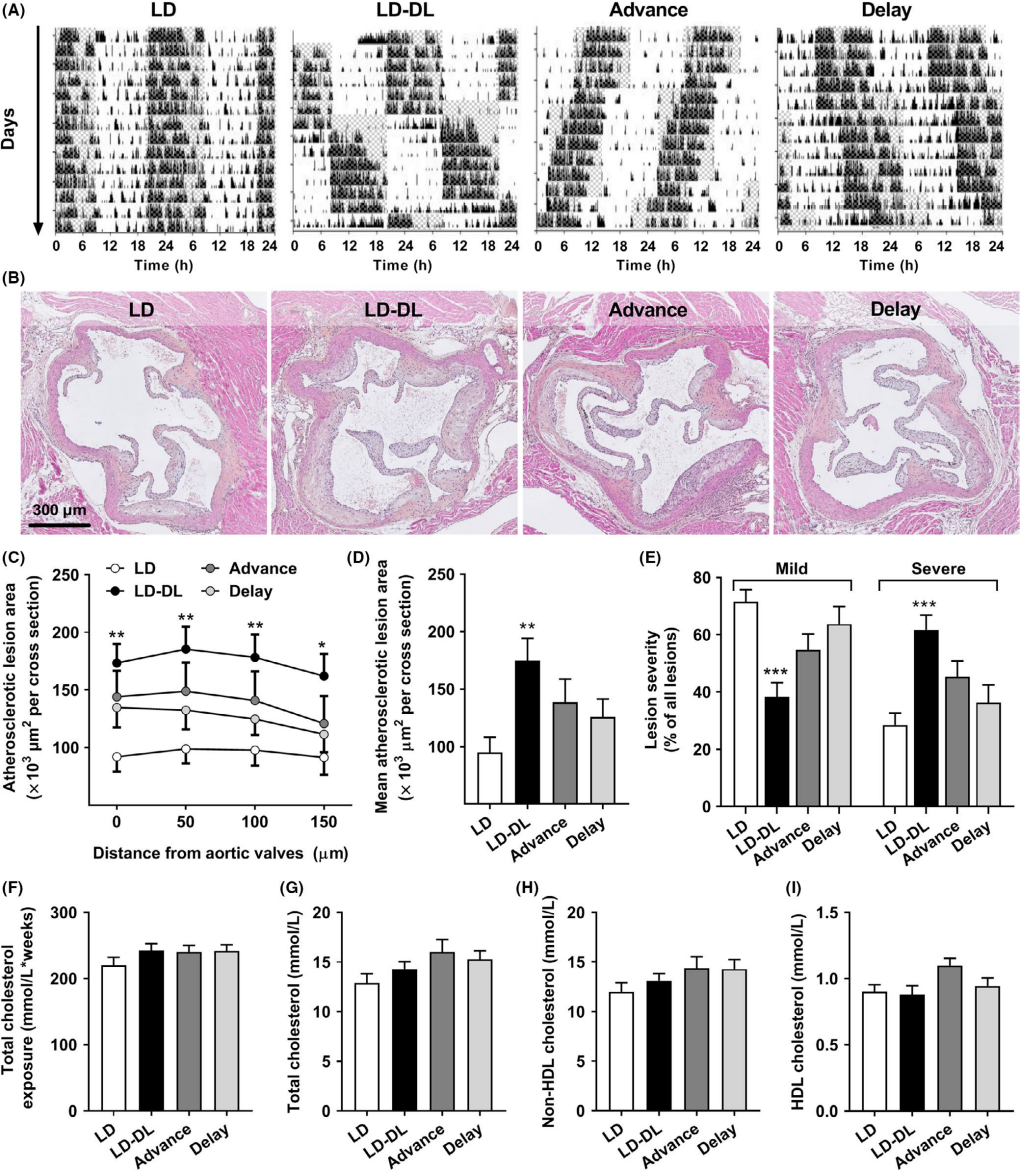
Weekly light shifts increase atherosclerosis development, without affecting plasma cholesterol. APOE*3‐Leiden.CETP mice (n = 15/group) were exposed to either regular light‐dark cycles (LD), weekly alternating light‐dark cycles (12 h shifts; LD‐DL), weekly 6 h phase advances (Advance), or weekly 6 h phase delays (Delay). (A) During weeks 14 and 15 of the intervention, mice were individually housed and behavioral activity was monitored by passive infrared monitors. Representative double‐plotted actograms are shown in which gray shading indicates the dark period. (B) After 15 wk, mice were sacrificed, hearts were isolated and sectioned, and sections of the valve area of the aortic root were stained with hematoxylin‐phloxine‐saffron (HPS). (C) Lesion area as a function of distance was determined, starting from the appearance of open valve leaflets covering 150 µm. (D) The mean atherosclerotic lesion area was determined from the four cross‐sections, (E) and lesion severity (mild, type I‐III vs severe, and type IV‐V) was scored. (F) Plasma cholesterol was determined at regular intervals to calculate total cholesterol exposure. (G) Total cholesterol, (H) non‐HDL‐cholesterol, and (I) HDL‐cholesterol were determined at end point (after 15 wk). Data represent means ± SEM. *P < .05, **P < .01, and ***P < .001 compared with the LD control group, according to one‐ or two‐way ANOVA

APOE*3‐Leiden.CETP mice, a well‐established mouse model for human lipoprotein metabolism and atherosclerosis, were subjected to weekly shifts in the light‐dark cycle to disrupt circadian rhythm, as illustrated by actograms in Figure 1A. Behavioral analysis of activity rhythms was performed to evaluate rhythm strength per day. Particularly, the LD‐DL group, which was subjected to 12 hours shifts in light‐dark cycle, demonstrated circadian disruption following a shift in light‐dark cycle, while weekly 6 hours phase advances and phase delays had a relatively mild effect on activity rhythms (Figure S2). After 15 weeks of light schedule interventions, we measured atherosclerosis development in the aortic root area. The LD‐DL group displayed a striking increase in atherosclerotic lesion area throughout the aortic root (Figure 1B,C), resulting in an almost twofold increased mean atherosclerotic lesion area (Figure 1D). Mice exposed to phase advances and delays did not show a significant increase in atherosclerosis development. The increased plaque size in the LD‐DL group was accompanied by a shift in plaque severity, as LD‐DL mice displayed a 47% decrease in mild lesions (types I‐III) and a 117% increase in severe lesions (types IV‐V) compared with controls (Figure 1E). The increased atherosclerotic lesion area in LD‐DL mice could also be observed in the whole aorta (Figure S3), although lesion development was much more pronounced in the aortic root.

Next, we evaluated whether weekly light shifts aggravated atherosclerosis development through changes in energy status or lipid metabolism. During the first 3 weeks of the study, weekly phase delays and alternating light‐dark cycles mildly reduced food intake (Figure S4A), however, this difference did not persist throughout the study. Body weight did not differ between experimental groups throughout the whole study (Figure S4B). Total unfasted plasma cholesterol was also similar between the groups at all time points measured (Figure S4C), resulting in an equal total cholesterol exposure in all groups (Figure 1F), as determined by calculating an area under the curve of all individual plasma cholesterol measurements shown in Figure S4C. At end point, after 15 weeks of light interventions, unfasted plasma levels of total cholesterol (Figure 1G), non‐HDL‐cholesterol (Figure 1H) and HDL‐cholesterol (Figure 1I), and the weight of metabolic organs (liver, interscapular brown adipose tissue [iBAT], and gonadal white adipose tissue [gWAT]; Figure S4D) were the same in all experimental groups.

### 3.2 Weekly light shifts increase lesion macrophages, but do not increase monocyte activation or ex vivo migration

**Figure 2:**
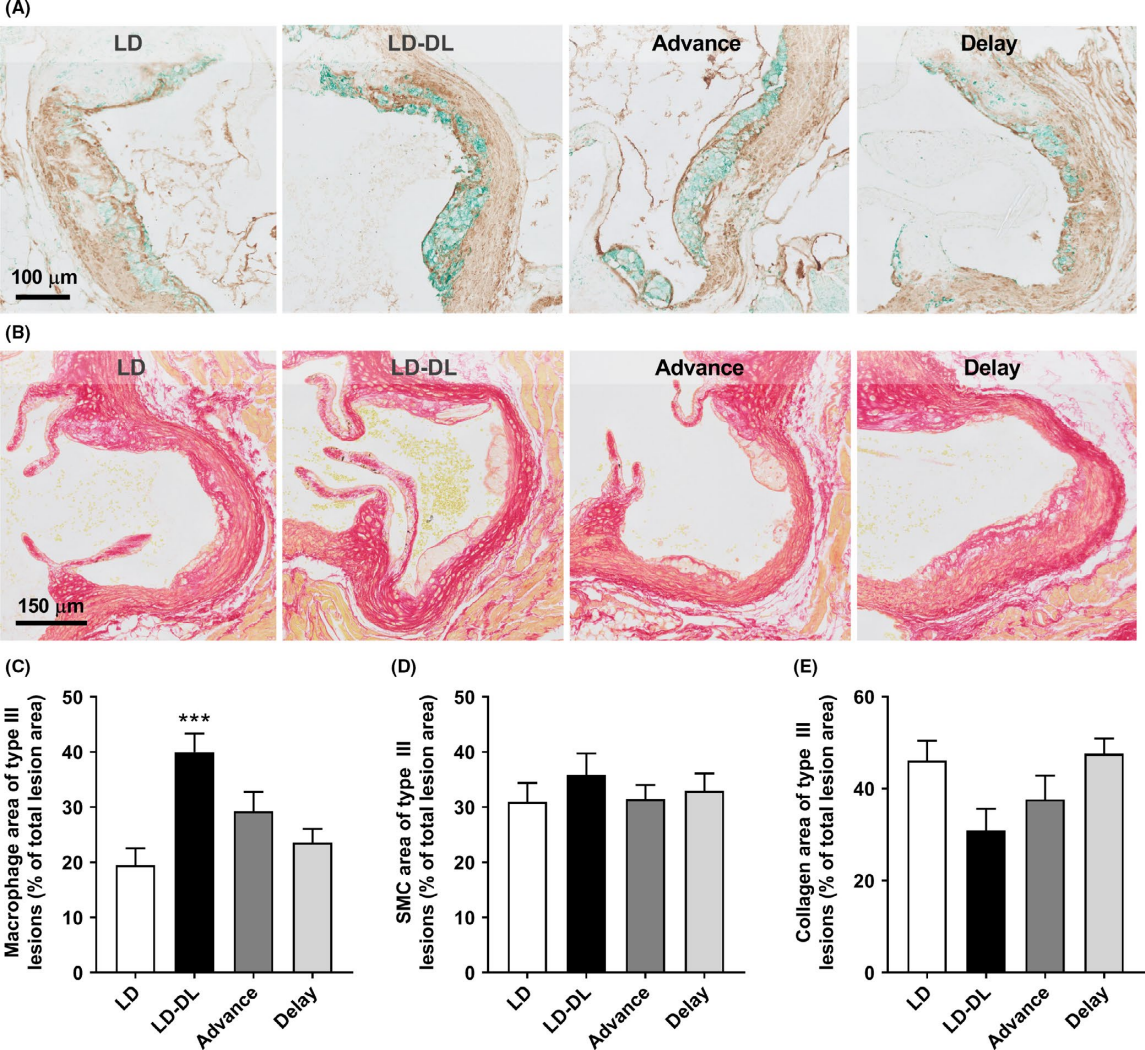
Weekly light shifts increase lesion macrophage content. APOE*3‐Leiden.CETP mice were exposed to either regular light‐dark cycles (LD), weekly alternating light‐dark cycles (12 h shifts; LD‐DL), weekly 6 h phase advances (Advance), or weekly 6 h phase delays (Delay) (n = 15/group). After 15 wk, mice were sacrificed, and hearts were isolated and sectioned. Slides of the valve area of the aortic root were double‐stained for (A) macrophages (MAC‐3; stained green) and smooth muscle cells (SMCs, actin; stained brown), and stained for (B) collagen with Sirius Red. The area of (C) macrophages, (D) SMCs, and (E) collagen of type III lesions was measured. Data represent means ± SEM. *P < .05 and ***P < .001 compared with the LD control group, according to one‐way ANOVA

To further identify what caused the aggravated atherosclerosis development in mice exposed to weekly shifts in the light‐dark cycle, we performed stainings for macrophages, smooth muscle cells (SMCs), collagen, and T cells and quantified the content for all intermediate type III lesions, as they contain all of these lesion components. In addition, by selecting one lesion type we controlled for the difference in lesion severity between groups (Figure 1E). In particular mice of the LD‐DL group showed an increase in lesional macrophage area (Figure 2A,C), while SMC area was not affected by shifts in light‐dark cycle (Figure 2A,D). Although we did not observe significant differences in collagen between groups, the collagen area did show an inverse pattern compared to the macrophage area, in line with studies showing that macrophages produce inflammatory factors such as metalloproteinases (MMPs) that degrade collagen and other extracellular matrix components,^21^ thereby reducing lesion stability. The number of Th and Tc cells within the atherosclerotic lesions was not affected by weekly alternating light‐dark cycles (Figure S5). Together, these data indicate that the aggravated atherosclerosis was driven by increased infiltration of monocytes in mice exposed to weekly light shifts.

**Figure 3:**
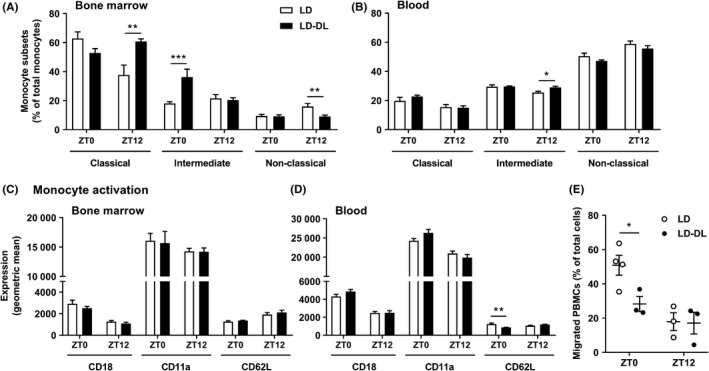
Weekly alternating light‐dark cycles do not increase monocyte activation or migration. APOE*3‐Leiden.CETP mice were exposed to either regular light‐dark cycles (LD) or weekly alternating light‐dark cycles (12 h shifts; LD‐DL) (n = 18/group) for 10 wk, after which they were sacrificed at either ZT0 or ZT12 (n = 9 per timepoint/group). Flow cytometry was used to analyze monocyte subsets (ie, classical [Ly6C^high^], intermediate [Ly6C^int^], and nonclassical [Ly6C^low^]) in (A) bone marrow and (B) blood and monocyte activation markers (ie*,* CD18, CD11a, and CD62L) in (C) bone marrow and (D) blood. (E) Peripheral blood mononuclear cells (PBMCs) were isolated from blood to study ex vivo migration toward the chemoattractant CCL2. PBMCs from multiple mice were pooled to result in a final n = 3 or 4/group. Data represent means ± SEM. *P < .05, **P < .01, and ***P < .001 compared with the indicated control group, according to two‐way ANOVA

To evaluate whether an increased monocyte infiltration could be the result of an increase in the number of circulating monocytes or other types of immune cells, we performed a second experiment in which mice exposed to either LD or LD‐DL were killed throughout the 3rd day in week 10 of the light intervention. Week 10 (instead of 15) was selected for these measurements as monocyte recruitment plays an important role particularly in early atherosclerosis development.^22^ Cosinor analysis revealed pronounced shifts in the oscillation of circulating leukocytes in LD‐DL mice, as defined by a difference in acrophase (circadian peak) between LD and LD‐DL mice (Figure S6). However, there was no effect on the total circulating amount of monocytes (Figure S6A‐C), Th cells (Figure S6D), Tc cells (Figure S6E), or B cells (Figure S6F). Also, we did not observe effects of the light intervention on the total amount of unfasted plasma cholesterol over a 24‐hours period (Figure S7). We further evaluated monocyte subsets and activation status at ZT0 and ZT12. These time points were selected as recent studies demonstrated a peak in leukocyte recruitment and migration into atherosclerotic lesions during the transition from the active phase (ZT12‐24) to the resting phase (ZT0‐12).^23^, ^24^ Furthermore, we observed identical monocyte numbers at ZT0 and ZT12 (Figure S6A‐C), while T‐cell numbers were increased at ZT0 in LD‐DL mice (Figure S6D,E), which could affect polarization and activation of monocytes. While monocyte precursors within the bone marrow did show a more pro‐inflammatory phenotype (ie, more classical [Ly6C^high^] and intermediate monocytes [Ly6C^int^], and less nonclassical monocytes [Ly6C^low^]) in the LD‐DL groups (Figure 3A), within the circulation we only observed a minor increase in intermediate monocytes (Figure 3B). To determine whether these monocytes could be more active, we measured surface expression of activity markers CD18 (integrin β2), CD11a (integrin αL), and CD62L (L‐selectin). These markers were unchanged in bone marrow of mice exposed to LD‐DL compared with LD (Figure 3C), and CD62L was even slightly reduced in the circulation of these mice at ZT0 (Figure 3D). In line with this, ex vivo migration of PBMCs (consisting of lymphocytes and monocytes) was not increased after 10 weeks of alternating light‐dark cycles (Figure 3E).

### 3.3 Weekly light shifts result in a more pro‐inflammatory vessel wall with increased expression of the chemokine CCL2

**Figure 4:**
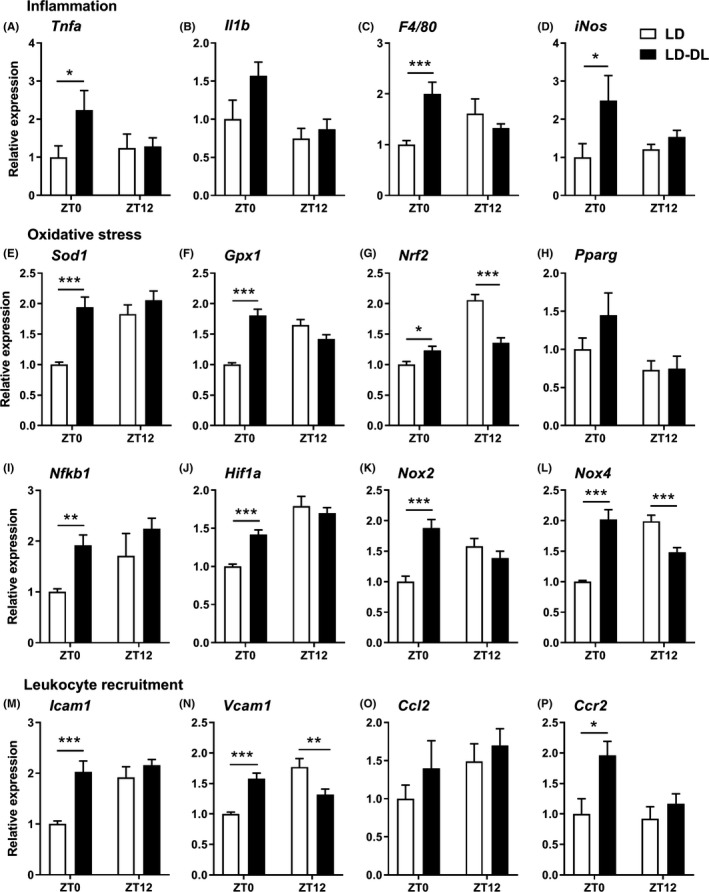
Weekly alternating light‐dark cycles increase gene expression of markers of inflammation, oxidative stress, and leukocyte recruitment within the aortic vessel wall. APOE*3‐Leiden.CETP mice were exposed to either regular light‐dark cycles (LD) or weekly alternating light‐dark cycles (12 h shifts; LD‐DL) (n = 18/group) for 10 wk, after which they were sacrificed at either ZT0 or ZT12 (n = 9 per timepoint/group). Aortas were isolated, and gene expression of markers of (A‐D) inflammation, (E‐L) oxidative stress, and (M‐P) leukocyte recruitment was measured by qRT‐PCR. Data represent means ± SEM. *P < .05, **P < .01, and ***P < .001 compared with the indicated control group, according to two‐way ANOVA

As the monocytes themselves did not seem to have an increased migratory capacity, we evaluated whether changes in the vessel wall could be underlying the increased monocyte infiltration and macrophage content within the atherosclerotic lesions. To this end, we examined gene expression of markers for inflammation (Figure 4A‐D), oxidative stress (Figure 4E‐L), and leukocyte recruitment (Figure 4M‐P) within the aortic vessel wall of mice exposed to either LD or LD‐DL for 10 weeks. At this moment, lesion development is still in an early stage, but the expression of inflammatory markers *Tnfa* (Figure 4A), *F4/80* (Figure 4C), and *iNos* (Figure 4D) was increased at ZT0 in LD‐DL mice. Additionally, expression of the oxidative stress genes *Sod1* (Figure 4E), *Gpx1* (Figure 4F), *Nrf2* (Figure 4G), *Nfkb1* (Figure 4I), *Hif1a* (Figure 4J), *Nox2* (Figure 4K), and *Nox4* (Figure 4L) was increased at ZT0, while *Nrf2* and *Nox4* expression was decreased at ZT12. Expression of the leukocyte adhesion molecules *Icam1* (Figure 4M), *Vcam1* (Figure 4N), and the chemoattractant receptor *Ccr2* (Figure 4P) was increased at ZT0, although *Vcam1* was similarly decreased at ZT12.

**Figure 5:**
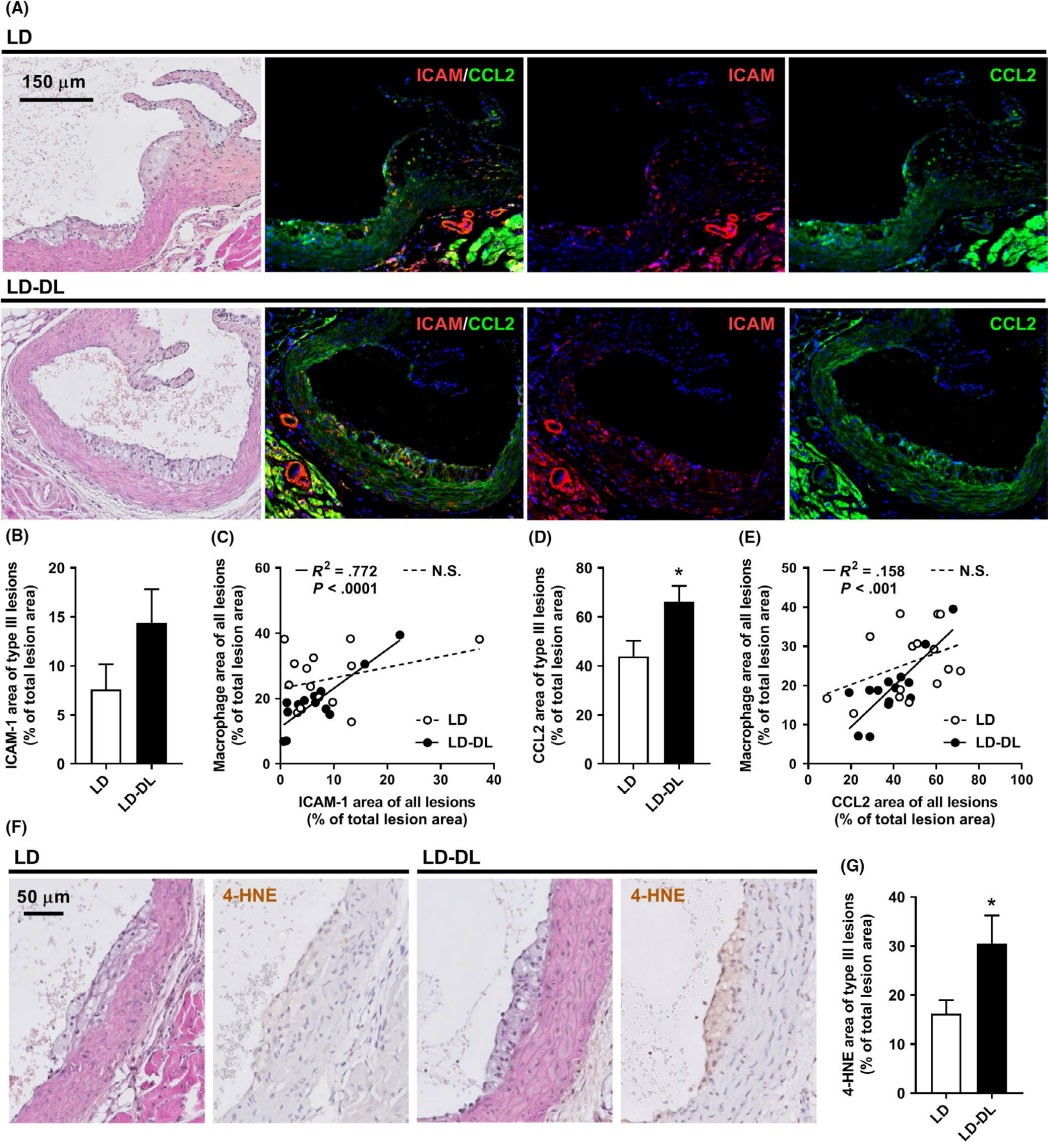
Weekly alternating light‐dark cycles increase CCL2 expression within atherosclerotic lesions. APOE*3‐Leiden.CETP mice were exposed to either regular light‐dark cycles (LD) or weekly alternating light‐dark cycles (12 h shifts; LD‐DL) (n = 15/group) for 15 wk, after which mice were sacrificed, hearts were isolated, and a double‐staining of ICAM‐1 and CCL2 was performed on sections of the aortic root. (A) Representative pictures show lesion areas in LD and LD‐DL mice stained with hematoxylin‐phloxine‐saffron (HPS) and double‐stained for ICAM‐1 and CCL2 (stained red and green, respectively) and counterstained with DAPI (blue). (B) ICAM‐1 area was determined within type III lesions, (C) and the relationship between ICAM‐1 and macrophage area was evaluated by Pearson correlation analysis. (D) CCL2 area was also determined within type III lesions and (E) correlated to macrophage area. Solid lines in the correlation plots indicate correlations within the LD‐DL group, and dashed lines indicate correlations within the LD group. (F) Representative pictures showing lesion areas in LD and LD‐DL mice stained with HPS and stained for 4‐hydroxynonenal (4‐HNE) and counterstained with hematoxylin. (G) 4‐HNE area was determined within type III lesions. NS, nonsignificant. Data represent means ± SEM. *P < .05 compared with the LD control group, according to the two‐tailed unpaired Student's *t* test

These gene expression results point toward a more inflamed and chemotactic phenotype of the vessel wall in mice exposed to shifts in light‐dark cycle. However, the observed gene expression effects could also have resulted from a shift in acrophase. Therefore, we aimed to substantiate these findings by evaluating protein expression specifically within atherosclerotic lesions after 15 weeks of intervention and performing correlation analyses with lesional macrophages. Immunofluorescent double‐staining for ICAM‐1 and CCL2 (Figure 5A) revealed a nonsignificant increase in ICAM‐1 area of type III lesions in LD‐DL mice compared with LD mice (Figure 5B). Specifically within the LD‐DL group, the amount of ICAM‐1 correlated positively with the macrophage content of the lesions (Figure 5C), consistent with the function of ICAM‐1 to facilitate leukocyte adhesion to the endothelium. CCL2, a chemokine that recruits monocytes to sites of endothelial injury by promoting endothelial transmigration, was markedly increased in type III lesions of LD‐DL mice (Figure 5D) and correlated strongly to the total lesion macrophage area within the LD‐DL group (Figure 5E). The presence of 4‐hydroxynonenal (4‐HNE), a product of lipid peroxidation and biomarker of oxidative stress, was evaluated through immunohistochemical staining (Figure 5F). Consistent with the observed increase in oxidative stress genes, 4‐HNE area was markedly higher in LD‐DL mice as compared to LD mice (Figure 5G).

## 4 DISCUSSION

In this study, we aimed to investigate whether mistimed light exposure has an effect on atherosclerosis development. We subjected APOE*3‐Leiden.CETP mice to weekly shifts in light‐dark cycle and observed a striking increase in atherosclerosis development. A complete reversal of the light and dark cycle (12 hours shifts) resulted in the strongest lesion progression, with an approximately twofold increase in atherosclerotic lesion size and severity. This demonstrates a causal relationship between mistimed light exposure and atherosclerosis.

In addition to mice subjected to 12 hours shifts in the light‐dark cycle, we studied mice that were subjected to 6 hours phase advances and 6 hours phase delays. We investigated these different light schedules as previous studies have shown that mice have more trouble adjusting to phase advances compared with phase delays,^25^, ^26^ resulting in a longer period of rhythm disturbance. However, we did not observe significant differences in adaptation between phase advances and phase delays, nor did these phase shifts result in a significant increase in atherosclerosis.

As most metabolic processes have a strong circadian rhythm, and circadian disruption results in metabolic alterations,^27^ we hypothesized that shifts in light‐dark cycle could aggravate atherosclerosis by dysregulating lipid metabolism. However, we did not observe any metabolic changes, which could explain such a large increase in atherosclerosis in mice exposed to light shifts. There have been previous studies investigating effects of shifts in light‐dark cycle on metabolic parameters in rodents,^28^ Some of these studies report an increased body weight in animals exposed to shifts in light‐dark cycle,^29^, ^30^ while others report no effect,^31^, ^32^ similar to our study. Of note, those studies mainly focused on glucose metabolism, while effects of rhythm disruption by shifts in light‐dark cycle on lipid metabolism have not been well‐studied. Nevertheless, in our study we did not find an indication that circadian disruption by alternating light‐dark cycles could affect lipid metabolism.

As the immune system is another main contributor of CVD, which is strongly regulated by the circadian clock,^15^, ^17^ we next focused on characterizing the inflammatory status of our mice. A recent study showed that circadian disruption by sleep fragmentation accelerates atherosclerosis development by increasing the number of circulating monocytes.^33^ However, in our study, there was no effect of circadian disruption by alternating light‐dark cycles on the total number of circulating immune cells throughout the day. Mice exposed to alternating light‐dark cycles did show shifts in oscillatory immune cell profiles (ie, of monocytes, T cells, and B cells). Potentially, mismatches between the oscillating leukocytes and the circadian environment could result in an altered immune cell function. Additionally, monocyte precursors within the bone marrow of these mice showed a more pro‐inflammatory phenotype, indicating that the myeloid immune system is becoming activated. Although this likely did not affect atherosclerosis development in our study as these changes in bone marrow were not yet accompanied by increased activation of circulating monocytes, it does suggest that a longer duration of light‐dark shifts could result in systemic myeloid activation and inflammation.

Furthermore, mice exposed to alternating light‐dark cycles showed an increase in vascular inflammation and oxidative stress. This could simply reflect the increased number of inflammatory cells within vessel wall, supported by increased expression of the macrophage marker *F4/80*. Nevertheless, this raises the question of how these cells have accumulated within the vessel wall, particularly, as the monocytes did not show an increased migratory capacity ex vivo. It is likely that through disturbance of cell‐extrinsic mediators of circadian rhythm (eg, glucocorticoid hormone or the sympathetic nervous system), alternating light‐dark cycles disrupted the core molecular clock of endothelial cells. Subsequently, changes in components of the molecular clock can affect mediators of inflammation and oxidative stress.^34^, ^35^ Our data show similar expression patterns of oxidative stress markers and *Icam1* and *Vcam1*, in line with previous studies showing that pro‐inflammatory cytokines can promote the expression of adhesion molecules such as ICAM‐1 and VCAM‐1 through induction of oxidative stress.^36^, ^37^ However, we did not find the same circadian organization in the expression of inflammatory cytokines (ie, *Tnfa* and *Il1b*), making this explanation less likely. Instead, disturbance of the molecular biological clock in endothelial cells can directly result in increased ICAM‐1 and VCAM‐1 expression, which has been shown in vitro,^38^ thereby promoting mononuclear cell adhesion to endothelial cells. This could result in the increased presence of monocytes in the vessel wall that notably expresses markers of inflammation and oxidative stress. Thus, expression of ICAM‐1 and VCAM‐1 may precede expression of inflammatory and oxidative stress genes.

Moreover, inflammation and oxidative stress have been shown to induce expression of CCL2,^39^ a chemokine that actively recruits monocytes to sites of endothelial injury. Although we did not find a significant difference in *Ccl2* gene expression in the aorta, we did observe increased CCL2 protein expression within atherosclerotic lesions of mice exposed to alternating light‐dark cycles. The CCL2‐CCR2 axis has been shown to play a major role in atherosclerosis. Genetic deletion of either CCL2 or CCR2 in both *Apoe* ^−/−^ and *Ldlr* ^−/−^ mice reduces the size of atherosclerotic lesions.^40^, ^41^, ^42^ In line with this, CCL2 overexpression aggravates atherosclerosis in *Apoe* ^−/−^ mice,^43^ and it has been suggested that CCL2 also drives disease progression in humans.^44^ Recently, it was shown that CCL2 expression within the vessel wall is rhythmic and drives rhythmic homing of monocytes to atherosclerotic lesions.^24^ Our results suggest that disturbing this rhythm could result in continuously high CCL2 expression within atherosclerotic lesions, increased monocyte migration, and atherosclerotic disease progression.

There have been previous studies examining a direct relationship between the biological clock and the atherosclerosis, by using male and female mice in which clock genes (ie, essential genes regulating the molecular biological clock) have been disrupted or augmented. Mutation of the *Clock* gene accelerates atherosclerosis in *Apoe* ^−/−^ and *Ldlr* ^−/−^ mice,^45^ while overexpression of the *Cry1* gene protects *Apoe* ^−/−^ mice from atherosclerosis development.^46^ These mouse models show significant alterations in cholesterol metabolism and inflammatory state, both likely contributing to the observed phenotype. Furthermore, hematopoietic *Rev‐erbα* knockdown increases atherosclerosis,^47^ and stimulation of REV‐ERB by agonist treatment reduces atherosclerosis in *Ldlr* ^−/−^ mice.^48^ In both studies, no effect on plasma cholesterol was observed, but changes in atherosclerosis rather depended on modulation of immune function, similar to our study. Although these studies also demonstrate a direct relationship between circadian rhythm and CVD, the models used do not mimic human shift work. In our study, we disrupted circadian rhythm by exposing mice to chronically alternating light‐dark cycles. This is a model of shift work, which has been used before to show an effect of rhythm disturbance on, for example, metabolic disease^49^ and cancer.^30^ In addition, we used APOE*3‐Leiden.CETP mice, a transgenic mouse model with a more human‐like lipoprotein metabolism compared with the commonly used *Apoe* ^−/−^ and *Ldlr* ^−/−^ mice.^18^, ^50^ In contrast to other mouse models of atherosclerosis, APOE*3‐Leiden.CETP mice respond similarly to medications used to prevent CVD as compared to humans.^51^, ^52^, ^53^ Thus, by selecting the current study setup and mouse model, we aimed to increase the translatability to the human shift work situation. It should be noted, however, that only female APOE*3‐Leiden.CETP mice develop hypercholesterolemia and atherosclerosis upon feeding a cholesterol‐rich diet. This is a limitation of the study as the immune response in atherosclerosis is differentially regulated in women and men.^54^ As there are also sex differences in human circadian rhythm,^55^ it would be very interesting for future studies to compare effects of rhythm disturbance on atherosclerosis development between males and females.

To conclude, we demonstrate for the first time that shifts in light‐dark cycle directly contribute to atherosclerosis development. Mechanistically, we show that disruption of circadian rhythm results in dysfunctional endothelium, with increased expression of cytokines, chemokines, and adhesion molecules. These changes in the vascular wall likely lead to an increased migration of monocytes from the circulation into atherosclerotic lesions, thereby increasing lesion macrophage content. These results demonstrate the detrimental effects of mistimed light exposure, as occurs in shift work, for the development of CVD. As the number of shift workers will not likely decrease in the future, novel strategies to improve circadian rhythm and vascular health in shift workers are of great importance.

## CONFLICT OF INTEREST

The authors declare that they have no conflict of interest.

## AUTHOR CONTRIBUTIONS

MS, RB, PCNR, and SK designed the experiments, with the help from LAB, BWO, NAMS, TG, DB, DV, JMG, EL, and NRB. MS, RB, LAB, BWO, NAMS, TG, LK, MRV, DV, and JMG performed experiments and analyzed data. METD, JFPB, JHM, KW, and LWMK provided intellectual contributions throughout the project. MS and SK wrote the manuscript. All authors critically reviewed the manuscript. PCNR and SK were responsible for the overall supervision of the study.
