# Supplementary material for: Disruption of circadian rhythm by alternating light‐dark cycles aggravates atherosclerosis development in APOE*3‐Leiden.CETP mice
Source: J Pineal Res. 2019 Oct 10;68(1):e12614. doi: 10.1111/jpi.12614 (PMC6916424; doi:10.1111/jpi.12614)
Supplement: Supplementary file 1 [file JPI-68-na-s001.docx]

**Supporting Information**

**Disruption of circadian rhythm by alternating light-dark cycles aggravates atherosclerosis development in APOE*3-Leiden.CETP mice**

Maaike Schilperoort, Rosa van den Berg, Laura A. Bosmans, Bram W. van Os, Martijn E.T. Dollé, Noortje A.M. Smits, Teun Guichelaar, Debbie van Baarle, Lotte Koemans, Jimmy F.P. Berbée, Tom Deboer, Johanna H. Meijer, Margreet R. de Vries, Dianne Vreeken, Janine M. van Gils, Ko Willems van Dijk, Linda W.M. van Kerkhof, Esther Lutgens, Nienke R. Biermasz, Patrick C.N. Rensen, Sander Kooijman

**Supplementary Materials and Methods**

**Plasma lipid measurements**

On indicated study days at Zeitgeber Time (ZT) 4 (i.e. 4 hours after onset of the light phase), unfasted blood was collected from the tail vein of mice into heparin-coated capillaries. Plasma was isolated by centrifugation, and plasma total cholesterol levels were measured by using an enzymatic kit (Roche Diagnostics). High-density lipoprotein (HDL) was isolated by precipitation of ApoB-containing lipoproteins. Hereto, 20% polyethylene glycol (Sigma-Aldrich) in 200 mM glycine-buffered saline (pH 10) was added to plasma (1:2, v/v) and centrifuged for 30 min at 6,000 rpm. HDL-cholesterol was determined by measuring total cholesterol in the supernatant as described above. Non-HDL-cholesterol was calculated by subtracting HDL-cholesterol from total cholesterol in plasma. Unfasted blood was collected from the tail vein every 4 hours over a 24-h period to evaluate daily variation in plasma total cholesterol levels.

**Behavioral analysis**

During week 14 and 15 of the light intervention, behavioral activity patterns were assessed by housing mice individually in cages fitted with passive infrared detectors. Behavioral patterns were analyzed by plotting actograms using ClockLab data analysis software (Actimetrics). The nocturnality index (activity in the dark phase versus the light phase) was calculated using activity data of week 14, to evaluate adaptation of activity rhythms per day.

**Histological and immunohistochemical analysis of the heart**

Hearts were fixated in 4% formalin, embedded in paraffin, and cross-sectioned (5 µm) throughout the aortic root area. Sections were stained with haematoxylin-phloxine-saffron (HPS) for histological analysis. Per mouse, atherosclerosis was analyzed in the aortic root starting from the appearance of open aortic valve leaflets in four subsequent sections with 50 µm intervals. Lesion area was determined using ImageJ software (version 1.50). Lesion severity (mild; types I-III and severe; types IV-V) was scored according to the guidelines of the American Heart Association adapted for mice, as described previously (Zadelaar et al., 2006). A double staining was performed for smooth muscle cells (SMCs) and macrophages by using primary antibodies against actin (M0851, 1:400; Dako) and MAC-3 (#550292, 1:1000; BD Pharmingen) and secondary antibodies K4001 (Dako) and MP-7444 (Vector), respectively. Sirius red (Chroma) was used to stain for collagen. Dual ICAM-1 and CCL2 staining was performed by using primary antibodies SC-1511 (1:200) and SC-1784 (1:300) (Santa Cruz) and secondary antibodies A-31573 and A-11055 (Thermo Fisher), respectively. The lipid peroxidation product 4-hydroxynonenal (4-HNE) was performed using primary antibody Ab46545 (1:200; Abcam) and secondary antibody P0448 (Dako). Dual CD3 and CD8 staining was performed by using primary antibodies MCA1477 (1:100; AbD Serotec) and 14–0808 (1:100; eBioscience) in combination with Vector Blue and Vector Red (Vector), respectively.

**Histological analysis of the aorta**

Whole aortas (from above the aortic root until 2-3 mm below the iliac bifurcation) were harvested, fixated in 4% formalin, and dissected from surrounding adipose tissue in Petri dishes layered with black dissecting wax (VWR, 470006-956). After cleaning, the aortas were cut open along the inner curvature of the aortic arch and pinned down using minutien pins (0.2 mm, Fine Science Tools, 26002-20). The pinned aortas were fixated overnight in 4% formalin, and stained in a 0.5% Oil Red O solution (Sigma-Aldrich, 00625) in 60% isopropanol for 30 min. Images of the aortas were taken at 10-fold magnification, and Oil Red O-positive lesion area was determined using ImageJ software (version 1.50).

**Flow cytometry**

In a second experiment, in week 10 of the light intervention, when light regimes were aligned among the groups, unfasted blood was collected via the tail vein in heparin tubes every 4 hours (ZT0, 4, 8, 12, 16 and 20) on the 3^rd^ day after a switch in light regime (*n* = 8 per timepoint/group), to evaluate circadian patterns of circulating white blood cells by flow cytometry. To determine the absolute number of immune cells per µl whole blood, cells were stained with fluorescently labeled surface antibodies (CD3, CD11b, CD115, Ly6C, NK1.1 from Biolegend, CD4 and CD8a from Pharmingen, CD19 from BD Horizon) in a staining solution (2% FCS in PBS), incubated with BD FACS Lysing Solution (BD Bioscience), and dyed with Flow-Count Fluorospheres (Becton Coulter), followed by analysis with a BD LSRFortessa X-20 flow cytometer (BD Biosciences).

**Monocyte subsets, activation status and migration**

To evaluate monocyte subsets and activation status, mice were sacrificed in week 10 of the light intervention on the 3^rd^ day after a switch in light regime at ZT0 and ZT12 (*n* = 9 per timepoint / group), followed by blood drawing via cardiac puncture with a syringe containing EDTA. Femurs were dissected to isolate bone marrow, and erythrocytes in the blood and bone marrow samples were lysed. Cells were stained in FACS buffer (0.5% BSA and 0.074% EDTA in PBS, pH set to 7.4-7.6) with fluorescently labeled surface antibodies (all from BD Biosciences: CD11a, CD11b, CD18, CD45, CD62L, CD115, cKit, Flt3, Ly6C), and analyzed with a BD Canto II flow cytometer (BD Biosciences). For the migration assay, peripheral blood mononuclear cell (PBMCs) were isolated from blood samples using a Ficoll gradient, with an additional 10 min incubation of the samples with red blood cell lysis buffer (ThermoFisher, 00-4300-54) to ensure the removal of all remaining erythrocytes. Chemotaxis of PBMCs towards 10 ng/ml recombinant mouse chemokine (C-C motif) ligand 2 (CCL2 or monocyte chemoattractant protein-1 (MCP-1); R&D systems, 479-JE) was evaluated in 96-well Boyden chambers with a 5 µm pore size filter (Neuroprobe, 106-5), and coated with 10 µg/ml fibronectin (Sigma, F4759). Cells from 1-3 mice were pooled and 0.1*10^6^ cells were applied to each filter in duplicate. Cells were allowed to migrate for 2 hours, after which cells in control wells and the migrated cells were counted in randomly selected fields.

**Gene expression analysis of aorta**

The upper parts of the aortas (from the heart to the diaphragm) were isolated and dissected from remaining surrounding tissue in RNA*later* RNA stabilization reagent (Qiagen). Subsequently, RNA was extracted by using TRIzol RNA isolation reagent (Thermo Fisher) following manufacturer’s protocol. RNA concentration was determined with a NanoDrop spectrophotometer (Thermo Fisher), and 100 ng RNA was transcribed with M-MLV Reverse Transcriptase (Promega). The qRT-PCR was performed using a SYBR Green kit (Promega) on a 7500 Fast RT-PCR System (Applied Biosystems). Primer sequences are listed in Table S1. mRNA expression of genes of interest was normalized to mRNA expression of the housekeeping genes *B2m* and *Gapdh*.

**References**

Zadelaar, A. S., Boesten, L. S., Jukema, J. W., van Vlijmen, B. J., Kooistra, T., Emeis, J. J., Lundholm, E., Camejo, G., & Havekes, L. M. (2006). Dual PPARalpha/gamma agonist tesaglitazar reduces atherosclerosis in insulin-resistant and hypercholesterolemic ApoE*3Leiden mice. *Arterioscler Thromb Vasc Biol, 26*(11), 2560-2566. doi:10.1161/01.Atv.0000242904.34700.66

**Supplementary Tables and Figures**

**Table S1. Primer Sequences for qRT-PCR**

| **Gene** | **Primer sequence** | | **Product length (bp)** |
| --- | --- | --- | --- |
| *B2m* | Forward  Reverse | 5’– TGACCGGCTTGTATGCTATC –3’  5’– CAGTGTGAGCCAGGATATAG –3’ | 222 |
| *Ccl2* | Forward  Reverse | 5’– AGCTGTAGTTTTTGTCACCAAGC –3’  5’– TGTCTGGACCCATTCCTTCTTG –3’ | 70 |
| *Ccr2* | Forward  Reverse | 5’– TGCCATCATAAAGGAGCCA –3’  5’– AGCACATGTGGTGAATCCAA –3’ | 91 |
| *F4/80* | Forward  Reverse | 5’– CTTTGGCTATGGGCTTCCAGTC –3’  5’– GCAAGGAGGACAGAGTTTATCGTG –3’ | 165 |
| *Gapdh* | Forward  Reverse | 5’– GGGGCTGGCATTGCTCTCAA –3’  5’– TTGCTCAGTGTCCTTGCTGGGG –3’ | 157 |
| *Gpx1* | Forward  Reverse | 5’– GGTTCGAGCCCAATTTTACA –3’  5’– CATTCCGCAGGAAGGTAAAG –3’ | 84 |
| *Hif1a* | Forward  Reverse | 5’– ACGAGAAGAAAAATAGGATGAGTTC –3’  5’– GTGGCAACTGATGAGCAAGC –3’ | 117 |
| *Icam1* | Forward  Reverse | 5’– TCCGCTGTGCTTTGAGAACT –3’  5’– TCCGGAAACGAATACACGGT –3’ | 75 |
| *Il1b* | Forward  Reverse | 5’– GCAACTGTTCCTGAACTCAACT –3’  5’– ATCTTTTGGGGTCCGTCAACT –3’ | 89 |
| *iNos* | Forward  Reverse | 5’– CGGGCATCTGGTAGCCAGCG –3’  5’– TGGCAACATCAGGTCGGCCAT –3’ | 110 |
| *Nfkb1* | Forward  Reverse | 5’– ACTGGAAGCACGGATGACAG –3’  5’– TCTCTGTCTGTGAGTTGCCG –3’ | 120 |
| *Nox2* | Forward  Reverse | 5’– CGAAAACTCCTTGGGTCAGC –3’  5’– GATTTCGACACACTGGCAGC –3’ | 97 |
| *Nox4* | Forward  Reverse | 5’– CCAAATGTTGGGCGATTGTGT –3’  5’– CAGGACTGTCCGGCACATAG –3’ | 96 |
| *Nrf2* | Forward  Reverse | 5’– GCAACTCCAGAAGGAACAGG –3’  5’– GGAATGTCTCTGCCAAAAGC –3’ | 203 |
| *Pparg* | Forward  Reverse | 5’– GTGCCAGTTTCGATCCGTAGA –3’  5’– GGCCAGCATCGTGTAGATGA –3’ | 142 |
| *Sod1* | Forward  Reverse | 5’– TACACAAGGCTGTACCAGTGC –3’  5’– ACATGCCTCTCTTCATCCGC –3’ | 84 |
| *Tnfa* | Forward  Reverse | 5’– AGCCCACGTCGTAGCAAACCAC –3’  5’– TCGGGGCAGCCTTGTCCCTT –3’ | 174 |
| *Vcam1* | Forward  Reverse | 5’– TGGAGGTCTACTCATTCCCTGA –3’  5’– GACAGGTCTCCCATGCACAA –3’ | 71 |

**
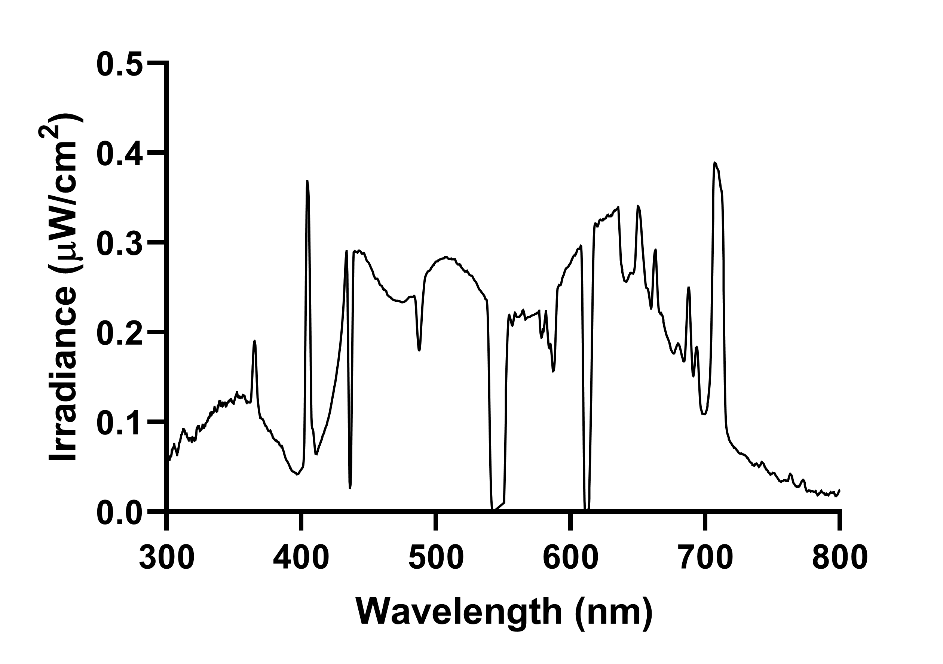
**

**Figure S1. Spectral power distribution of the light source.** APOE*3-Leiden.CETP mice were housed in light-tight cabinets fitted with diffuse white fluorescent light (50-100 lux). Spectral power distribution was measured with an AvaSpec 2048-SPU (Avantus BV, The Netherlands) light meter.

**
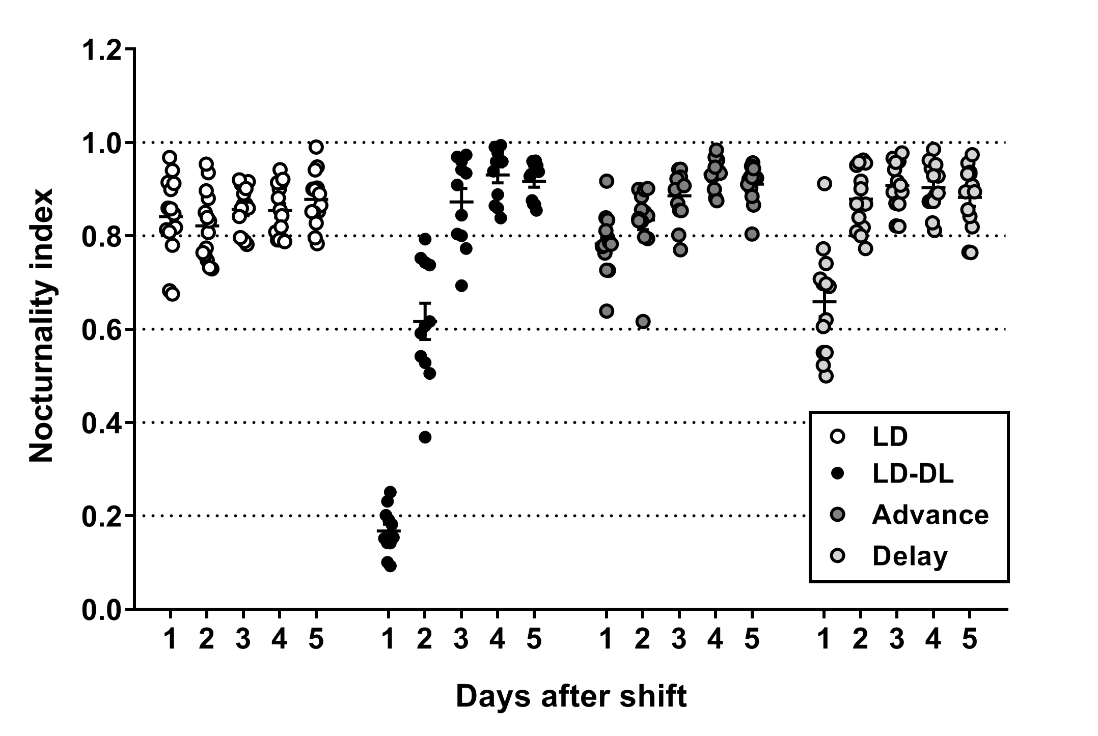
**

**Figure S2. Shifts in light-dark cycle disrupt circadian activity rhythm.** APOE*3-Leiden.CETP mice (*n* = 15/group) were exposed to either regular light-dark cycles (LD), weekly alternating light-dark cycles (12 h shifts; LD-DL), weekly 6 h phase advances (Advance) or weekly 6 h phase delays (Delay) for 15 weeks. Behavioral activity data of week 14 of the study was used to calculate the nocturnality index (activity in the dark versus light phase), to evaluate rhythm strength per day after a shift in light-dark cycle. The figures shows individual data points, including group means ± SEM.

**
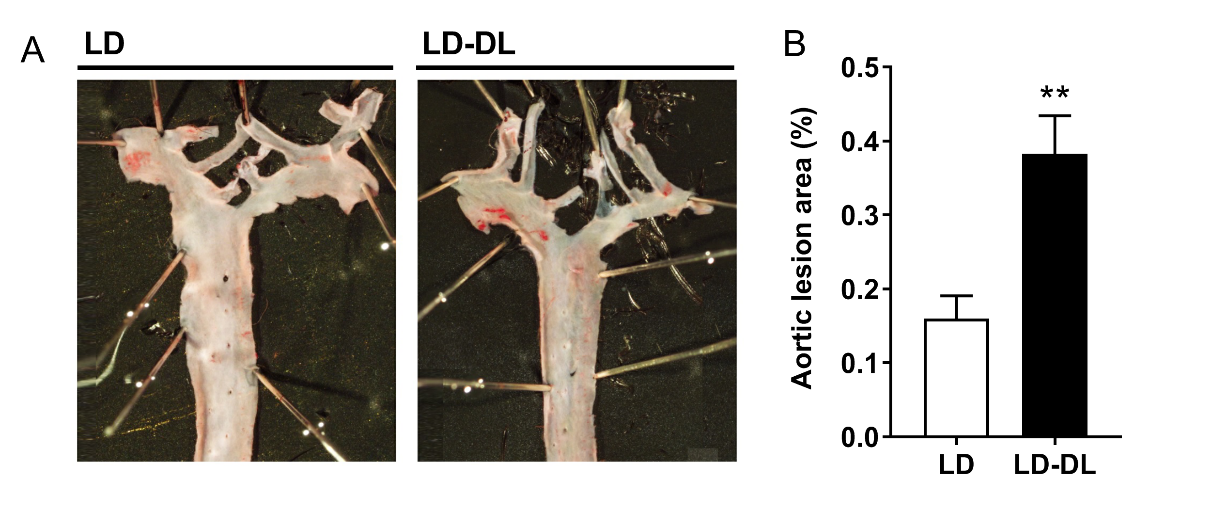
**

**Figure S3. Weekly light shifts increase atherosclerosis development in the aorta.** APOE*3-Leiden.CETP mice (*n* = 7/group) were exposed to either regular light-dark cycles (LD) or weekly alternating light-dark cycles (12 h shifts; LD-DL) for 15 weeks, after which mice were sacrificed, aortas were isolated, and an Oil Red O staining was performed on the whole aorta. (**A**) Representative images of the ascending aorta, aortic arch and part of the descending aorta stained with Oil-Red-O. (**B**) The Oil-Red-O-positive atherosclerotic lesion area was quantified in the whole aorta. Data represent means ± SEM. ***P* < 0.01 compared to the LD control group, according to the two-tailed unpaired Student T test.

**
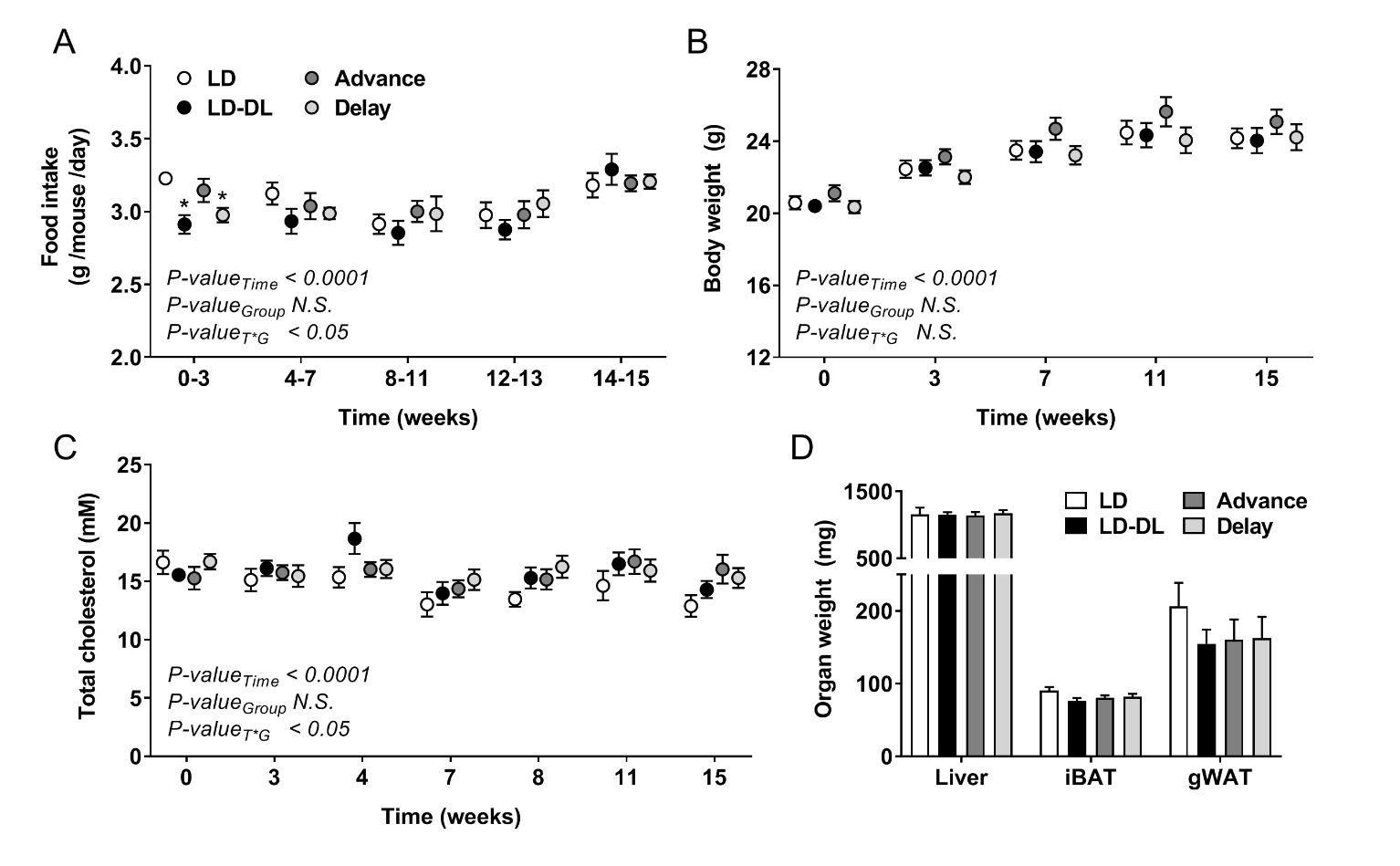
 Figure S4. Weekly light shifts do not affect metabolic parameters.** APOE*3-Leiden.CETP mice (*n* = 15/group) were exposed to either regular light-dark cycles (LD), weekly alternating light-dark cycles (12 h shifts; LD-DL), weekly 6 h phase advances (Advance) or weekly 6 h phase delays (Delay). (**A**) Food intake, (**B**) body weight, and (**C**) plasma total cholesterol were determined at regular intervals. (**D**) After 15 weeks, mice were sacrificed and metabolic organs were weighed. Data represent means ± SEM. Group differences were determined by repeated measurement ANOVA with Dunnett’s post hoc test (A), mixed model ANOVA (B, C), or regular two-way ANOVA (D). *P*-values of the factors Time and Group are indicated, as well as the interaction between Time and Group (T*G) (A-C). **P <* 0.05 compared to the LD control group.


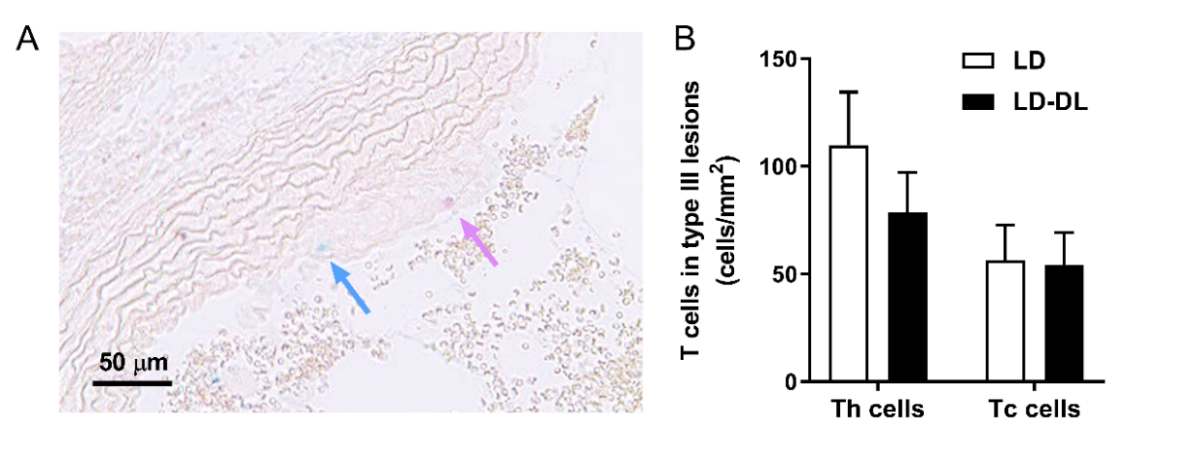


**Figure S5. Weekly alternating light-dark cycles do not increase lesion T cell counts.** APOE*3-Leiden.CETP mice (*n* = 15/group) were exposed to either regular light-dark cycles (LD) or weekly alternating light-dark cycles (12 h shifts; LD-DL) for 15 weeks, after which mice were sacrificed, hearts were isolated, and a double-staining of CD3 (blue) and CD8 (pink) was performed on sections of the aortic root (**A**; example of a type III lesion with a blue-stained CD3^+^CD8^-^ Th cell and a purple-stained CD3^+^CD8^+^ Tc cell, as indicated by the blue and purple arrows, respectively). (**B**) Th and Tc cells in type III lesions were counted. Data represent means ± SEM.


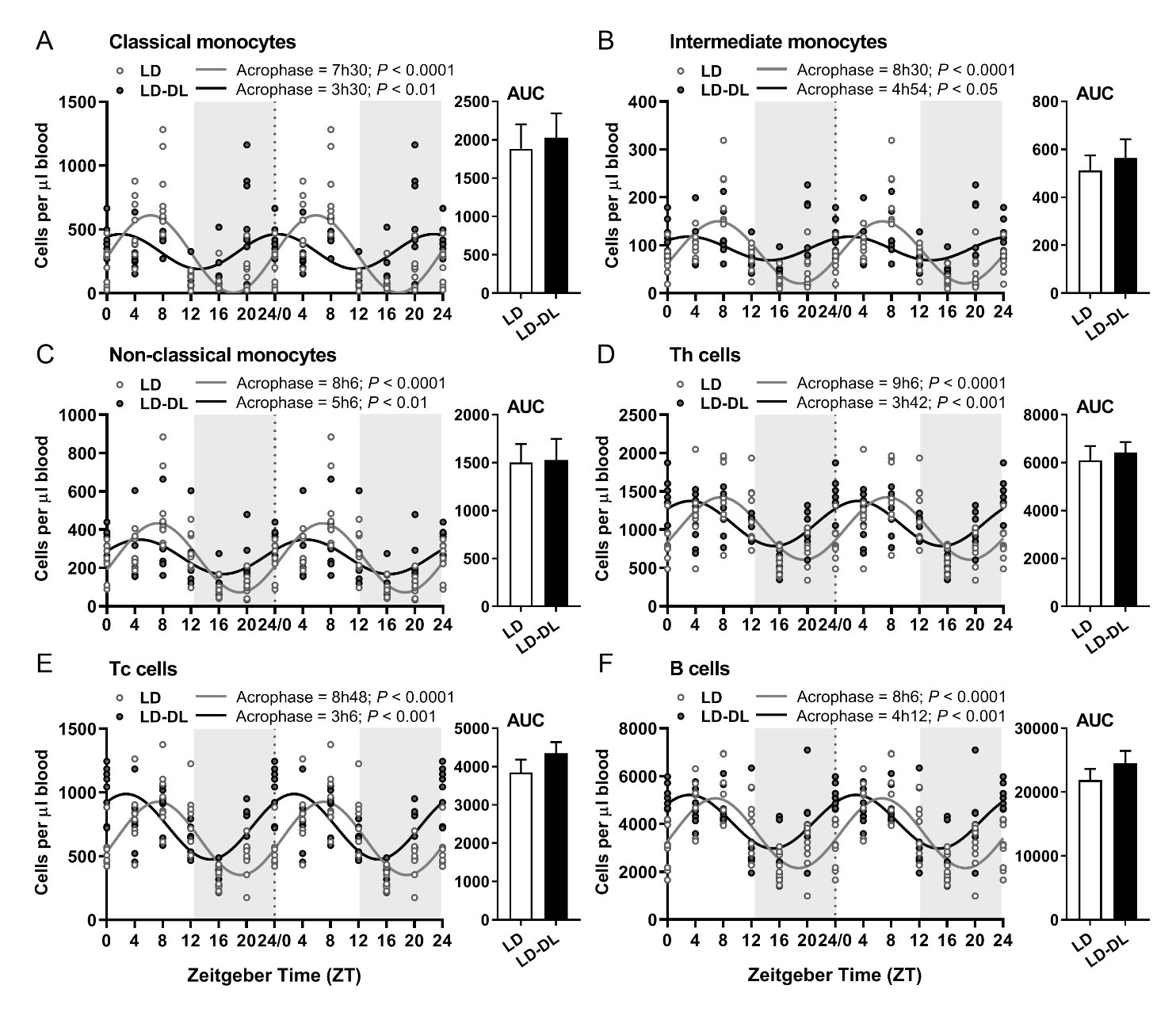


**Figure S6. Weekly alternating light-dark cycles do not affect the total amount of circulating immune cells.** Blood was drawn every 4 hours in APOE*3-Leiden.CETP mice (*n* = 8 per timepoint/group) exposed to 10 weeks of either regular light-dark cycles (LD) or weekly alternating light-dark cycles (12 h shifts; LD-DL), three days after a shift in light-dark cycle. The total amount of (**A**) classical, (**B**) intermediate and (**C**) non-classical monocytes, (**D**) Th cells, (**E**) Tc cells and (**F**) B cells was determined by flow cytometry. Data points were double plotted from the dotted lines, and an area under the curve (AUC) was calculated over a 24 h period. Light and grey areas represent the light and dark phase, respectively. Data represent means ± SEM. Cosinor analysis was performed to determine the acrophase and corresponding *P*-values for both groups.

**
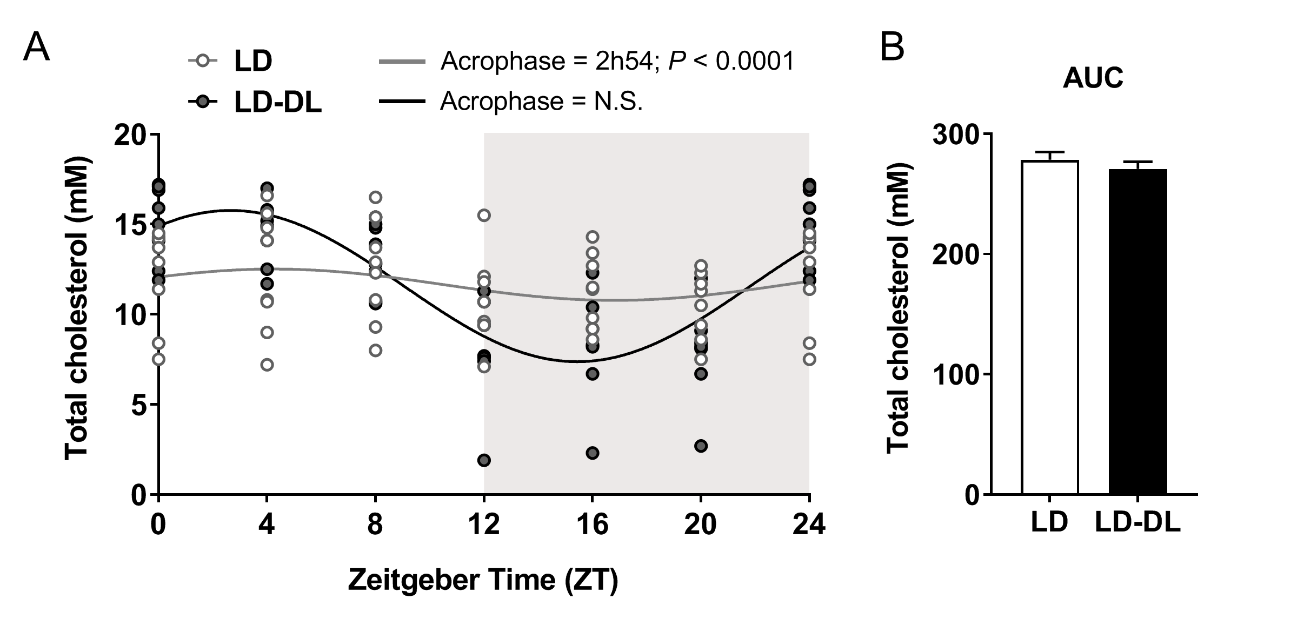
**

**Figure S7. Weekly alternating light-dark cycles do not affect the total amount of plasma cholesterol.** Blood was drawn every 4 hours in APOE*3-Leiden.CETP mice (n = 8 per timepoint/group) exposed to 10 weeks of either regular light-dark cycles (LD) or weekly alternating light-dark cycles (12 h shifts; LD-DL), three days after a shift in light-dark cycle. (**A**) Total plasma cholesterol was measured at all timepoints, (**B**) and used to calculate an area under the curve (AUC) of total cholesterol. Light and grey areas represent the light and dark phase, respectively. Data represent means ± SEM. Cosinor analysis was performed to determine the acrophase and corresponding *P*-values for both groups.
